# Subcellular Dynamics of a Conserved Bacterial Polar Scaffold Protein

**DOI:** 10.3390/genes13020278

**Published:** 2022-01-30

**Authors:** Giacomo Giacomelli, Helge Feddersen, Feng Peng, Gustavo Benevides Martins, Manuela Grafemeyer, Fabian Meyer, Benjamin Mayer, Peter L. Graumann, Marc Bramkamp

**Affiliations:** 1 Institute for General Microbiology, Christian-Albrechts-University Kiel, Am Botanischen Garten 1-9, 24118 Kiel, Germany; ggiacomelli@ifam.uni-kiel.de (G.G.); hfeddersen@ifam.uni-kiel.de (H.F.); fpeng@ifam.uni-kiel.de (F.P.); mgrafemeyer@ifam.uni-kiel.de (M.G.); fmeyer@ifam.uni-kiel.de (F.M.); 2 Faculty of Biology, Ludwig-Maximilians-University Munich, Großhaderner Straße 2-4, 82152 Planegg-Martinsried, Germany; martinsbenevides@gmail.com; 3 SYNMIKRO, LOEWE-Zentrum für Synthetische Mikrobiologie, Karl von Frisch Straße 14, D-35043 Marburg, Germany; mayerb@staff.uni-marburg.de (B.M.); graumanp@uni-marburg.de (P.L.G.); 4 Fachbereich Chemie, Universität Marburg, Hans-Meerwein Strasse 4, D-35032 Marburg, Germany; 5 Central Microscopy Facility, Christian-Albrechts-University Kiel, Am Botanischen Garten 1-9, 24118 Kiel, Germany

**Keywords:** DivIVA, *Corynebacterium*, *Bacillus*, ParB, MinJ, single-molecule localization microscopy

## Abstract

In order to survive, bacterial cells rely on precise spatiotemporal organization and coordination of essential processes such as cell growth, chromosome segregation, and cell division. Given the general lack of organelles, most bacteria are forced to depend on alternative localization mechanisms, such as, for example, geometrical cues. DivIVA proteins are widely distributed in mainly Gram-positive bacteria and were shown to bind the membrane, typically in regions of strong negative curvature, such as the cell poles and division septa. Here, they have been shown to be involved in a multitude of processes: from apical cell growth and chromosome segregation in actinobacteria to sporulation and inhibition of division re-initiation in firmicutes. Structural analyses revealed that DivIVA proteins can form oligomeric assemblies that constitute a scaffold for recruitment of other proteins. However, it remained unclear whether interaction with partner proteins influences DivIVA dynamics. Using structured illumination microscopy (SIM), single-particle tracking (SPT) microscopy, and fluorescent recovery after photobleaching (FRAP) experiments, we show that DivIVA from *Corynebacterium glutamicum* is mobilized by its binding partner ParB. In contrast, we show that the interaction between *Bacillus subtilis* DivIVA and its partner protein MinJ reduces DivIVA mobility. Furthermore, we show that the loss of the rod-shape leads to an increase in DivIVA dynamics in both organisms. Taken together, our study reveals the modulation of the polar scaffold protein by protein interactors and cell morphology. We reason that this leads to a very simple, yet robust way for actinobacteria to maintain polar growth and their rod-shape. In *B. subtilis*, however, the DivIVA protein is tailored towards a more dynamic function that allows quick relocalization from poles to septa upon division.

## 1. Introduction

Stable inheritance of genetic material is a prerequisite for life. Therefore, all cells have established sophisticated processes for precise organization and segregation of their genetic material. Most bacterial cells contain one or few chromosomes that are spatially well organized. In recent years, the subcellular organization of bacterial chromosomes was analyzed in different model species. A common scheme is that both arms of the mainly circular chromosomes reside in defined cellular areas [1,2,3]. The localization of the origin of replication (*oriC*) is found in two main organizations. Some bacteria, such as *Escherichia coli* or vegetative growing *Bacillus subtilis*, replicate their DNA from a central position and push the replicated *oriC* regions towards the cell poles [4]. In contrast, *Caulobacter crescentus* has a polar localized *oriC* that is stably tethered to the stalked cell pole [5]. During DNA replication, one of the newly replicated origins is segregated across the existing nucleoid towards the other pole [5,6]. The functional connection of cell morphology and genome organization is a fundamental process of cellular self-organization, but the molecular details of these processes are not fully understood.

In many bacteria, segregation of origin regions is regulated by ParABS systems. Originally identified as plasmid segregation complexes [7], these DNA transport systems are also encoded on the main chromosome of various bacteria [8]. The general structure of these systems comprises three elements: two proteins (ParA and B) and a specific DNA motif (*parS*). ParB is a CTP-binding protein that binds specifically to *parS* sites [8,9,10,11,12]. These inverted repeats are clustered around the *oriC* region [8]. ParB dimers close around the DNA in a CTP-dependent fashion and spread from *parS* sites across the DNA [13,14]. Upon CTP hydrolysis, the ParB proteins are released from the DNA and can be loaded again. The localized loading and spreading and the CTP-mediated switch generate a zone of high ParB density around the *oriC* region that helps to structure the DNA. In vitro experiments suggest that ParB subunits form liquid-liquid phase separated condensate that may assist the segregation process in vivo [15]. The ParB–*parS* nucleoprotein complex is required for efficient *oriC* segregation. The directional movement of the cluster is achieved by the ParA ATPase. In their ATP-bound form, ParA dimers bind to DNA, not specifically. ATP hydrolysis is then triggered by ParB, releasing ParA from the DNA [15]. This interaction between ParA and ParB generates a diffusion ratchet-type segregation mechanism, in which ParB condensates follow the highest concentration of ParA [16]. Since ParA exploits the existing nucleosome as a spatial track, this type of DNA segregation is found in bacteria with a polarly tethered *oriC* region. Polar tethering is generally mediated by scaffold proteins that have been shown to oligomerize in a nucleotide independent fashion. In alpha-proteobacteria, such as *C. crescentus* or *Magnetospirillum gryphiswaldense*, polar tethering is mediated by PopZ [17,18]. PopZ is a soluble protein with a conserved N- and C-terminal region and a large unstructured central region. This structure allows oligomerization into large gel-like networks that localize ParB–*parS* complexes to the old cell poles [19,20].

In many Gram-positive bacteria, a coiled-coil protein termed DivIVA serves as the polar scaffold for *oriC* localization [21]. DivIVA binds to the plasma membrane through a N-terminal motif composed of a hydrophobic residue that is flanked by positively charged amino acids [22]. The central part of DivIVA is composed of coiled coils that can vary in length, depending on the organism. In particular, actinobacterial DivIVA proteins are larger and have extended coiled-coil regions [23]. The C-terminal part is essential for tetramerization of DivIVA. Lateral interactions of DivIVA tetramers lead to the formation of larger, lattice-like structures [22]. It was shown that DivIVA binds preferentially to curved membrane regions [23]. Therefore, DivIVA concentrates at the cell poles in rod-shaped cells. However, upon initiation of cell division, DivIVA quickly relocalizes to the invaginating septum, since the inward-growing septum displays a higher degree of membrane curvature [24,25,26]. 

The *B. subtilis* DivIVA (DivIVA*_bsu_*) is part of the Min system, a protein complex that spatially controls cytokinesis and ensures that cell division occurs only once per cell cycle. In vegetative cells, DivIVA*_bsu_* binds to MinJ and is enriched at the cell poles [27]. After the onset of cell division, MinJ and DivIVA*_bsu_* relocalize to the inward growing septum [26]. The dynamics of this system have been recently described and mathematically modeled [26]. However, during sporulation, DivIVA*_bsu_* is required as a polar scaffold for chromosome tethering. This is achieved by direct interaction with the DNA-binding protein RacA [28,29].

In *Corynebacterium glutamicum*, DivIVA (DivIVA*_cgb_*) is essential for the polar localization of the apical cell wall synthesis machinery [30]. DivIVA*_cgb_* recruits the elongation-specific transglycosylase RodA [31,32] and likely also Penicillin-Binding-Proteins [33]. DivIVA is an essential protein in actinobacteria, and DivIVA*_cgb_* depletion leads to loss of polar growth, resulting in a coccoid cell morphology [30]. Furthermore, we have shown before that DivIVA*_cgb_* also interacts with ParB, thereby tethering the *oriC* region to the cell pole [34]. The dual function of DivIVA*_cgb_* is likely the reason why the protein is essential in these bacteria [35]. In the closely related Mycobacteria, DivIVA (also termed Wag31) was also shown to interact with ParB and ParA proteins [36], while in the filamentous Streptomyces, DivIVA is coordinating apical growth and recruitment of the ParB-origin region [37]. Splitting of the polar DivIVA cluster leads to establishment of branches that grow out and result in the characteristic hyphal network, with similar behavior being observed also for non-branching organisms such as *C. glutamicum* [38]. Chromosome segregation into newly formed branches is simultaneously established via DivIVA-ParB interactions.

Despite the broad conservation and the essentiality of DivIVA proteins in many bacteria, little is known about the assembly and protein dynamics in vivo. Furthermore, DivIVA serves diverse functions in different species, and therefore, a molecular adaptation to its specific role seems likely. The DivIVA superfamily has previously been divided into two subgroups, one for actinobacteria and one for firmicutes. We therefore set out to characterize the precise localization and single-molecule dynamics of DivIVA in two species, each representative for one of the two main DivIVA subgroups: *C. glutamicum* and *B. subtilis*. Our data reveal that the single-molecule dynamics of DivIVA proteins match precisely their cellular roles, and the DivIVA dynamics are modulated by their interaction partners. We also analyzed the effect of the cell geometry on the single-molecule behavior of DivIVA proteins. We show here that DivIVA dynamics are directly influenced by the cell shape and that strong negative curvature has the distinct effect of slowing down DivIVA mobility. Thus, DivIVA assembly at cell poles is facilitated by the cell’s geometry, and hence, is a simple but efficient way for cellular self-organization. Stabilizing the DivIVA assemblies at the cell poles allows for robust maintenance of the rod-shaped morphology. In turn, de novo formation of a polarity axis by assembly of DivIVA in regions of increased curvature after loss of rod-shape morphology is self-stabilized, thereby quickly allowing the establishment of a new rod-shaped cell geometry.

## 2. Materials and Methods

### 2.1. Cloning

All oligonucleotides, plasmids and strains used in this study are listed in Tables S1–S3, respectively. All molecular clonings were performed using *E. coli* NEB5α (Table S3). Correct plasmid construction was controlled by DNA sequencing.

For the construction of pk19mobsacB-divIVA-HaloTag, HaloTag was amplified from pJet1.2 Tev-Halo tag plasmid DNA via PCR with the SalI-5´Halo-Fwd and XbaI-TAA-3´Halo-Rev oligos (Table S1), digested with SalI and XbaI and ligated into SalI- and XbaI-digested pk19mobsacB-divIVA-mNeonGreen. 

The sgRNA sequence used for the depletion of DivIVA*_cgb_* (agccttctgagcggccttgg, site: 323–345) was obtained via the CHOPCHOP website (http://chopchop.cbu.uib.no/, accessed on 20 October 2020). Briefly, the pSGdCas9 plasmid [39] was digested with BsaI and ligated to the previously annealed sgRNA-divIVA-F and sgRNA-divIVA-R oligos (Table S1), obtaining pSGdCas9-sgRNA-DivIVA. 

pHF29 [pUC18mut-divIVA-mNG-aad9-divIVAdown] was constructed by a Golden Gate assembly of five fragments: (1) PCR with primers HF0061 and HF0062 using pUC18mut as template (yielding a linear pUC18mut; (2) PCR with primers G34 and G35 and 168 genomic DNA (containing the C-terminal region of divIVA); (3) PCR with primers HF0211 and HF0212 and pJet1.2 Tev-Halo tag plasmid DNA (containing the Halo gene); (4) PCR with primers G36 and G37 and BHF028 genomic DNA (containing the spectinomycin adenyltransferase aad9); (5) PCR with primers G32 and G33 and 168 genomic DNA (containing the region downstream of divIVA).

### 2.2. Strain Construction

Both the pk19mobsacB-divIVA-HaloTag and the pk19mobsacB-divIVA-mNeonGreen plasmids were transformed into *C. glutamicum* RES 167 derivatives (RES167 _IsceIrs) to obtain B6G8 (*C. glutamicum* RES167 *adhA*::*adhA*_sw-IsceI_rs, *divIVA*::*divIVA*-HaloTag) and B4B7 (*C. glutamicum* RES167 *adhA*::*adhA*_sw-IsceI_rs, *divIVA*::*divIVA*-mNeonGreen), respectively.

The pSGdCas9-sgRNA-DivIVA plasmid was transformed in B6G8 to obtain B6I1 (*C. glutamicum* RES167 *adhA*::*adhA*_sw-IsceI_rs, *divIVA*::*divIVA*-HaloTag, pSGdCas9-sgRNA-DivIVA).

The pEKEx2-ParB-CFP plasmid was transformed in B6G8 to obtain CMG029 (*C. glutamicum* RES167 *adhA*::*adhA*_sw-IsceI_rs, *divIVA*::*divIVA*-HaloTag, pEKEx2-ParB-CFP).

The pk19mobsacB-Δ*parB* plasmid was transformed in B6G8 to obtain CMG027 (*C. glutamicum* RES167 *adhA*::*adhA*_sw-IsceI_rs, *divIVA*::*divIVA*-HaloTag, Δ*parB*)

The pUC18mut-divIVA-Halo-aad9-divIVAdown plasmid was transformed into *B*. *subtilis* 168 and RD021 to obtain BHF073 (DivIVA-HaloTag) and BHF074 (DivIVA-HaloTag, Δ*minJ*), respectively.

### 2.3. Wide-Field Microscopy

*C. glutamicum* strains were grown at 30 °C, 200 rpm in BHI medium (Table S4) for overnight culture. The next day, cells were diluted to an OD_600_ of 0.3 in fresh BHI and grown for approximately 3 h (OD = 3). DivIVA was depleted (CPF005) by supplementing the bacterial culture with 0.2 mM IPTG three hours prior to harvesting.

### 2.4. HADA Staining

1 mL of bacterial culture was used to harvest the cells with Centrifuge (2 min, 12,000 rpm). The cells were resuspended in 25 µL of 1× PBS, and 0.25 µL of 5 mM HADA dissolved in DMSO was added (final concentration 1%). The cells were incubated at RT for 5 min in the dark. After centrifugation, the cells were washed two times (1 mL, 1× PBS) and resuspended in 1 mL of PBS. The cells (2 µL) were collected on the agarose glass slides and analyzed by phase contrast and fluorescence microscopy using CFP or DAPI channel (Carl Zeiss Axio Imager Microscope, Oberkochen, Germany).

### 2.5. Fluorescence Recovery after Photo-Bleaching (FRAP)

For FRAP experiments, a Delta Vision Elite imaging system (GE Healthcare, Applied Precision) equipped with an InsightSSI illumination unit, an X4 laser module, and a CoolSnap HQ2 charge-coupled device (CCD) camera was used. Images were taken with a 100× oil PSF U-Plan S-Apo 1.4 numerical aperture objective every 20 s on both the transmitted light (0.01 s exposure time, 100% transmittance) and mCherry (0.25 s exposure time, 32% transmittance) channels. Bleaching was performed using a 561 nm laser (50 mW) with 25% power and a 0.02 s pulse. 

Analysis of the images was performed using ImageJ 1.53 g as part of the Fiji package [40]. The corrected total cell fluorescence (CTCF) was calculated according to the following formula: CTCF = integrated density (area of selected cell × mean fluorescence of unspecific background readings) [41]. Unspecific background was subtracted for every region of interest (ROI) (see above). The CTCF of the polar DivIVA foci was divided by the CTCF of the whole cell to account for photobleaching during acquisition. Relative values were used in order to allow comparison between cells, where a value of 1 corresponds to the fluorescence intensity before the bleaching event, and a value of 0 corresponds to the fluorescence intensity 20 s after the bleaching event. This time point was chosen in order to exclude mCherry photoswitching from the determination of the half-time recovery [42].

To determine half-time recovery and mobile/immobile fractions, the FRAP curve from the normalized recovery values was fitted to an exponential equation:

(1) I(t)=A(1 − e−τt) 

where *I*(*t*) is the normalized FRAP curve, *A* is the final value of the recovery, *τ* is the fitted parameter, and *t* is the time after the bleaching event. After determination of the fitted coefficients, they can be used to determine mobile (*A*) and immobile (1 − *A*) fractions, while the following equation was used to determine halftime recovery:

(2) T1/2=ln 0.5−τ

where *T*1/2 is the halftime recovery and *τ* is the fitted parameter. Graphs and statistics were run in R 3.3.1 [43,44], utilizing the packages ggplot2 [45] and nlstools [46]. 

### 2.6. Single-Molecule Localization Microscopy (SMLM) and Lattice Structured Illumination Microscopy (Lattice-SIM)

#### 2.6.1. *B. subtilis* Sample Preparation

*B. subtilis* was grown on nutrient agar plates using commercial nutrient broth and 1.5% (*w*/*v*) agar at 37 °C overnight. To reduce inhibitory effects, antibiotics were used only for transformations, since allelic replacement is stable after integration (spectinomycin, 100 μg mL^−1^).

For microscopy, *B. subtilis* was inoculated to an OD_600_ of around 0.05 from a fresh overnight culture or plate and grown in MD medium (Table S4), a modified version of Spizizen minimal medium at 30 °C with aeration in baffled shaking flasks (200 rpm) to an OD_600_ of 1. For the growth of protoplasts, MD medium was further supplemented with osmoprotective MSM medium (Table S4). Subsequently, cultures were diluted to an OD_600_ of 0.1 in fresh MD medium and grown to an OD_600_ of 0.5 (exponential phase). For staining, 1 mL of bacterial culture was moved to a 2 mL microcentrifuge tube and incubated with 5 nM HaloTag TMR ligand (SPT only) or 1 µM FM4-64 dye (SIM only) at 30 °C for 10 min. Protoplasts were stained during lysozyme treatment for only 10–20 min. Cells were harvested (4000 rpm, 3 min, 30 °C) and washed four times in MD medium, and in case of protoplasts, only twice with MD medium supplemented with MSM. Finally, TetraSpeck beads were added to the SIM samples to a final concentration of 1:1000.

#### 2.6.2. *B. subtilis* Protoplast Generation

*B. subtilis* strains were plated on nutrient agar (NA) plates and grown overnight. Multiple colonies were used to inoculate 10 mL MD medium and grown at 30 °C with aeration in baffled shaking flasks (200 rpm) to an OD_600_ of 1. Subsequently, cultures were diluted to an OD_600_ of 0.1 in fresh MD medium and grown to an OD_600_ of 0.5 (exponential phase). An amount of 2 mL of bacterial culture was moved to a 2 mL microcentrifuge tube and harvested (4000 rpm, 3 min, 30 °C) to replace the MD medium with fresh MD medium supplemented with MSM for osmoprotection. An amount of 4 mg mL^−1^ lysozyme was added, and cells were incubated for 10–20 min, until the formation of protoplasts was complete, which was confirmed microscopically. Cells were then harvested (4000 rpm, 3 min, 30 °C) and washed twice in MD/MSM buffer.

#### 2.6.3. *C. glutamicum* Sample Preparation

*C. glutamicum* cells, L-forms included, were grown at 30 °C, 200 rpm unless otherwise stated. Plates and liquid cultures were supplemented with 50 µg mL^−1^ kanamycin when needed (CDC013, CMG029, B6I1 and CPF005). Cells grown in osmoprotective L-form supporting medium (MSM)/CGXII [47] (Table S4).

*C. glutamicum* strains were plated on MSM/NA plates and grown at 30 °C overnight. Colonies were picked in the morning and resuspended in 10 mL of MSM/nutrient broth (NB) (Table S4). Cells were then grown over-day in baffled flasks. In the evening, 5 mL of cell suspension was moved to a new flask, supplemented with 5 mL of fresh MSM/CGXII medium and grown overnight. The next day, cells were diluted to an OD_600_ of 1 in MSM/CGXII and grown for approximately 3.5 h. An amount of 1 mL of bacterial culture was moved to a 2 mL microcentrifuge tube and incubated with 5 nM HaloTag TMR ligand (SPT only) for 30 min at 30 °C or 1 µM FM4-64 dye (SIM only) for 10 min at 30 °C. Cells were harvested (4000 rpm, 3 min, 30 °C) and washed four times in TSEMS osmoprotective buffer (Table S4). Finally, TetraSpeck beads were added to the SIM samples to a final concentration of 1:1000.

DivIVA was depleted (B6I1 and CPF005) by supplementing the bacterial culture with 0.2 mM IPTG three hours prior to harvesting.

ParB-CFP expression (CDC013 and CMG029) was induced by supplementing the bacterial culture with 0.1 mM IPTG two hours prior to harvesting.

#### 2.6.4. *C. glutamicum* L-Forms

*C. glutamicum* strains were plated on MSM/NA plates and grown overnight. Multiple colonies were transferred to an MSM/NA plate supplemented with D-cycloserine (DCS) (plates were coated with 500 µL of a 10 mg mL^−1^ D-cycloserine solution) and grown overnight. Cells were grown over-day (7 h) in 5 mL MSM/NB supplemented with 400 µg mL^−1^ DCS in a glass tube. An amount of 2.5 mL of L-forms suspension were then moved to a new tube, supplemented with 2.5 mL of fresh MSM/CGXII-glucose (DCS is consequently added to a final concentration of 400 µg mL^−1^) and grown for two days at 120 rpm. DCS was added daily to avoid reversion to rod-shape. 1 mL of bacterial culture was moved to a 2 mL microcentrifuge tube and incubated with 5 nM HaloTag TMR ligand for 30 min. Cells were harvested (4000 rpm, 3 min, 30 °C) and washed twice in TSEMS buffer.

#### 2.6.5. Slide Preparation 

Slides and coverslips were first cleaned by overnight storage in 1 M KOH, carefully rinsed with ddH_2_O, and subsequently dried with pressurized air. Next, 1% (*w*/*v*) low melting agarose (agarose, low gelling temperature, Sigma-Aldrich, Taufkirchen, Germany) was dissolved in the respective medium/buffer (MD or MD/MSM for *B. subtilis*/protoplasts, respectively, and TSEMS for *C. glutamicum*) for 1 h at 95 °C shaking. Medium/buffer were sterile filtered (0.2 µm pore size) shortly before being used to remove particles. To produce flat, uniform, and reproducible agarose pads, gene frames (Thermo Fisher, Dreieich, Germany) were utilized, and pads were finally allowed to solidify for 1 h at room temperature to be used within the next 3 h.

#### 2.6.6. SMLM Imaging

SMLM imaging was performed with an Elyra 7 (Zeiss) inverted microscope equipped with two pco.edge sCMOS 4.2 CL HS cameras (PCO AG), connected through a DuoLink (Zeiss), only one of which was used in this study. Cells were observed through an alpha Plan-Apochromat 63×/1.46 Oil Korr M27 Var2 objective in combination with an Optovar 1.6× (Zeiss) magnification changer, yielding a pixel size of 63 nm. During image acquisition, the focus was maintained with the help of a Definite Focus.2 system (Zeiss). Fluorescence was excited with a 561 nm (100 mW) laser, and signals were observed through a multiple beam splitter (405/488/561/641 nm) and laser block filters (405/488/561/641 nm) followed by a Duolink SR QUAD (Zeiss) filter module (secondary beam splitter: LP 560, emission filters: EF BP420-480 + BP495-550). 

Cells were illuminated with the 561 nm laser (50% intensity) in TIRF mode (62° angle). For each time lapse series, 10,000 frames were taken with either 5 ms exposure time (~9 ms with transfer time included) and 100% 561 nm intensity laser or 20 ms exposure time (~24 ms with transfer time included) and 50% 561 nm intensity laser. 

For single-particle tracking, spots were identified with the LoG Detector of TrackMate v6.0.1 [48], implemented in Fiji 1.53 g [42], an estimated diameter of 0.5 μm, and median filter and sub-pixel localization activated. The signal to noise threshold for the identification of the spots was respectively set at 7 for the 20 ms exposure time datasets and at 5 for the 5 ms exposure time datasets. To limit the detection of particles to single-molecules, frames belonging to the bleaching phase of TMR were removed from the time lapses prior to the identification of spots. Spots were merged into tracks via the Simple LAP Tracker of TrackMate, with a maximum linking distance of 500 nm, two frame gaps allowed, and a gap closing max distance of 800 nm. Only tracks with a minimum length of 5 frames were used for further analysis, yielding a minimum number of total tracks per sample of 825 for the depletion of DivIVA and 2216 for all other strains.

To identify differences in protein mobility and/or behavior, the resulting tracks were subjected to dwell time, mean-squared-displacement (MSD), and square displacement (SQD) analysis in SMTracker 2.0 [49], described previously [50].

Dwell time distribution was determined for a confinement radius of 97 nm and fitted with two components. The average MSD was calculated for four separate time points per strain (exposure of 20 ms—τ = 24, 48, 72, and 96 ms/exposure of 5 ms—τ = 9, 18, 27, and 36 ms), followed by fitting of the data to a linear equation. The last time point of each track was excluded to avoid track-ending-related artifacts. The cumulative probability distribution of the square displacements (SQD) was used to estimate the diffusion constants and relative fractions of up to three diffusive states. Diffusion constants were determined simultaneously for the compared conditions, therefore allowing for a more direct population fraction comparison.

#### 2.6.7. Lattice SIM Imaging

Lattice SIM imaging was performed with an Elyra 7 (Zeiss) inverted microscope equipped with two pco.edge sCMOS 4.2 CL HS cameras (PCO AG) connected through a DuoLink (Zeiss), both of which were used in this study. Cells were observed through an alpha Plan-Apochromat 63×/1.46 Oil Korr M27 Var2 objective in combination with an Optovar 1.6× (Zeiss) magnification changer, yielding a pixel size of 63 nm. Fluorescence was excited with a 488 nm (100 mW) laser, and signals were observed through a multiple beam splitter (405/488/561/641 nm) and laser block filters (405/488/561/641 nm) followed by a Duolink SR QUAD (Zeiss) filter module (secondary beam splitter: LP 560, emission filters: EF BP 420-480 + BP 495-550 and BP 570-620 + LP 655). 

Cells were illuminated with a 488 nm laser (50% intensity for *C. glutamicum* cells and 30% intensity for *B. subtilis* cells). The distance between Z-positions (330 nm) was optimized for Leap mode, a novel mode of acquisition and processing developed by Zeiss. Each Z-plane was imaged in Lattice-SIM mode and comprises 15 phases. For each phase, cells were imaged for 100 ms. Temperature was maintained at 30 °C for the entirety of the imaging.

Lattice SIM image reconstruction was performed via the ZEN 3.0 SR (black) software (Zeiss). Dual iterative SIM (SIM^2^), a nonlinear iterative reconstruction algorithm developed by Zeiss, was used for the image reconstruction. Reconstruction was performed for all Z-planes and fluorescent channels with 3D Leap processing active. SIM^2^ general settings were set to “Live” and “Strong” with the following specifics: 40 iterations, a regularization weight of 0.02, a processing sampling of 2×, and an output sampling of 2×. Advanced filter parameters were set to “Median” and “Best fit” with a sectioning value of 100. The obtained distance between two reconstructed Z-planes equals 110 nm.

The reconstructed Z-stacks obtained for the two cameras were aligned along the *Z*-axis via the comparison of the *Z*-axis point spread function (PSF) obtained for the TetraSpeck beads located within the same field of view. Finally, XY-alignment between the separate fluorescent channels was performed via the Channel alignment function within the ZEN 3.0 SR (black) software (Zeiss). Briefly, channel alignment mode was set to “Affine”, with active “Fit” and “Markers”. TetraSpeck beads were used as alignment markers (>3 markers for field of view).

#### 2.6.8. Statistics

Mean square displacement, jump distance, dwelling time analysis and related statistics were performed via SMTracker 2.0. FRAP-related statistics were performed via Rstudio (R studio version 1.4.1106, R version 4.0.5 (31 March 2021) [43,44]. The following packages were used: ggplot2 [45], PMCMRplus [51], and nlstools [46].

## 3. Results

### 3.1. Structure and Localization of DivIVA Differs between B. subtilis and C. glutamicum

**Figure 1:**
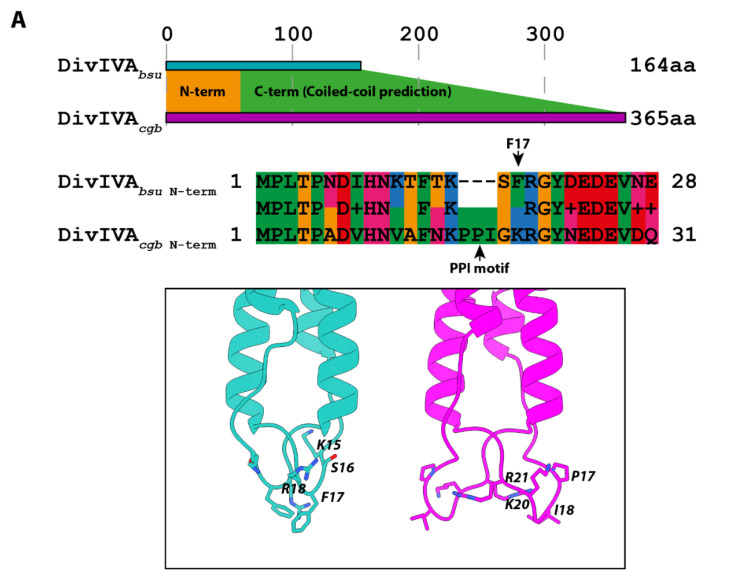

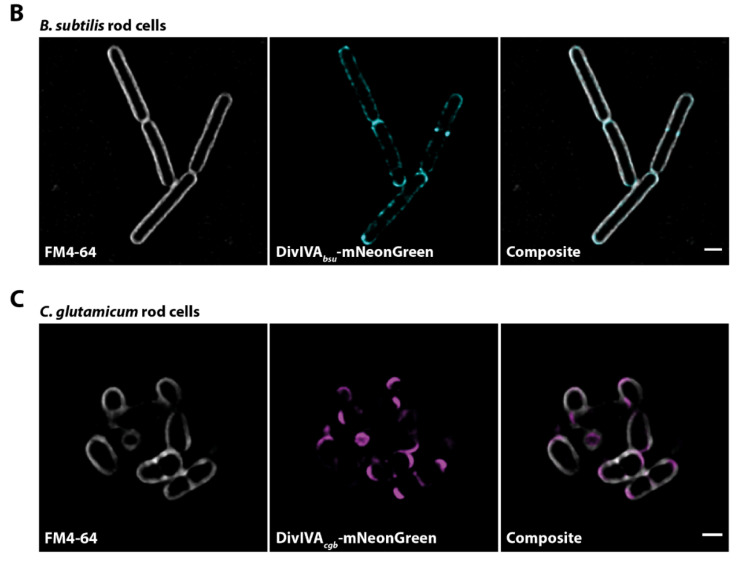
DivIVA localizes differently in *B. subtilis* and *C. glutamicum*. (**A**) Topology of DivIVA*_bsu_* and DivIVA*_cgb_*. The conserved N-terminal region responsible for membrane binding and protein dimerization is shown in orange, while the less conserved C-terminal coiled-coiled region responsible for oligomerization of DivIVA proteins is shown in green. This region is likely responsible for most of the functional differences across the DivIVA superfamily. The sequence alignment of the DivIVA N-terminal region responsible for membrane binding in *B. subtilis* and *C. glutamicum* follows the Lesk color scheme. Residues responsible for direct membrane interaction, F17, and P16P17I18, respectively, are indicated via black arrows. The corresponding 3D structure is shown in cyan for *B. subtilis* and in magenta for *C. glutamicum* (both structures are shown as dimers). Exponentially growing cells of *B. subtilis* (BHF028) (**B**) or *C. glutamicum* (B4B7) (**C**) expressing a DivIVA-mNeonGreen fusion were stained with FM4-64 membrane dye and imaged using structured illumination microscopy (SIM^2^ algorithm). From left to right: FM4-64 membrane dye, DivIVA-mNeonGreen, and composite of both channels. Scale bar 1 µm.

Previous BLAST searches with the protein sequence of *B. subtilis* DivIVA resulted in the identification of three major subgroups: a group comprising DivIVA proteins from the actinobacteria, one comprising DivIVA proteins from the firmicutes, and a final one comprising GpsB proteins from the firmicutes [52]. We selected a representative protein for each of the two main DivIVA proteins subgroups: DivIVA*_cgb_* (cg2361) for the actinobacteria and DivIVA*_bsu_* (BSU15420) for the firmicutes. Both representative proteins are characterized by the typical DivIVA domain architecture, with a conserved N-terminal domain responsible for lipid binding and dimerization and a less conserved C-terminal coiled-coiled domain required for oligomerization (Figure 1A) [22]. While the N-terminal domain is mainly conserved across the two subgroups, it has been previously shown that the conservation does not extend to the residues that are directly responsible for the interaction with the membrane. This function is, in fact, attributed to the phenylalanine 17 residue in the *B. subtilis* DivIVA protein (PDB code 2wuj; [22]) while it has been instead tentatively assigned to the structurally rigid P16P17I18 motif in *Mycobacterium tuberculosis* [53].

We first modeled the N-terminal region of DivIVA*_cgb_* (Figure 1A) with Wag31 (Rv2145c) as a template (PDB code 6lfa; [53]) and could confirm that the PPI membrane interface domain three-dimensional arrangement is conserved between *C. glutamicum* and *M. tuberculosis*.

Consistent with the presence of significant differences in sequence and structure between DivIVA*_bsu_* and DivIVA*_cgb_*, the SIM^2^ imaging of the respective mNeonGreen tagged versions revealed dissimilarities in their affinity to curvatures (Figure 1B,C). DivIVA*_bsu_* subunits formed clusters that associated with the lateral cell membrane but were more enriched at cellular regions characterized by a higher degree of negative curvature, such as poles and newly forming septa (Figure 1B). These regions contained a higher amount of protein in comparison to the lateral cell membrane, while never achieving a homogenous coverage of the cellular compartment. DivIVA*_cgb_* on the other side formed seemingly homogeneous structures that covered the quasi-entirety of the polar and septal regions, with little to no signal being visible along the lateral cell membrane (Figure 1C).

### 3.2. DivIVA_bsu_ Displays Increased Mobility and Decreased Dwelling Time Compared to DivIVA_cgb_

**Figure 2:**
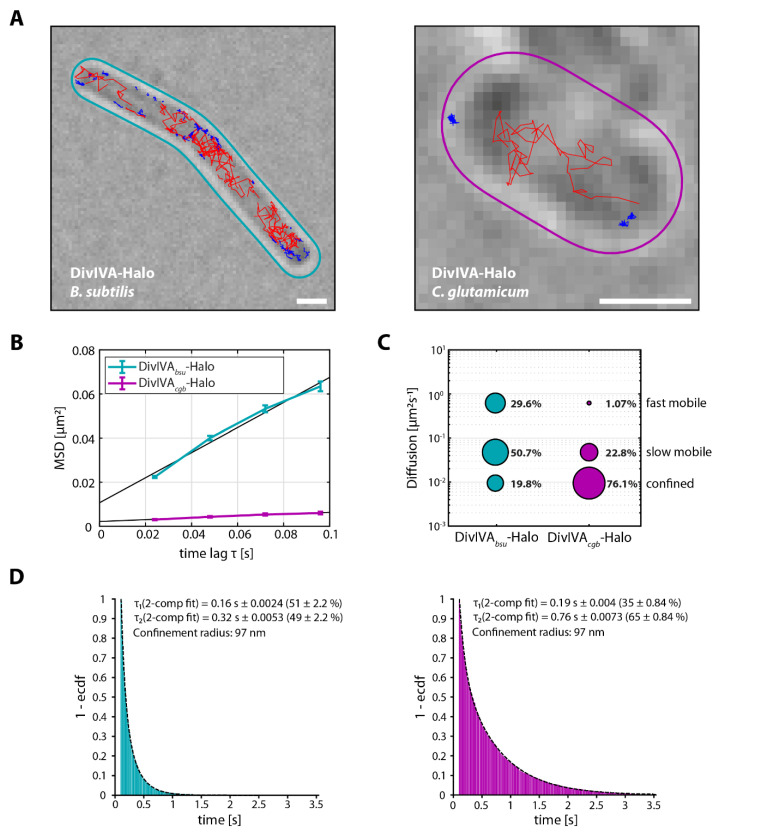

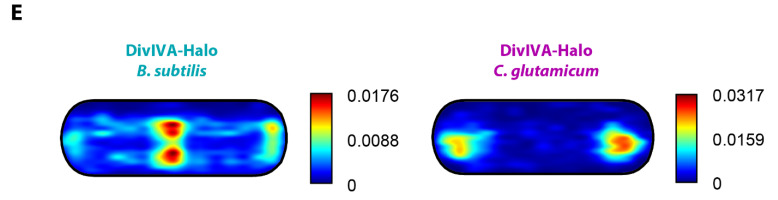
DivIVA*_bsu_* displays faster mobility and lower dwelling times compared to DivIVA*_cgb_*. Exponentially growing cells of *B. subtilis* (left/cyan panels) (BHF073) or *C. glutamicum* (right/magenta panels) (B6G8) expressing a DivIVA-Halo fusion, respectively, were stained with TMR (HaloTag ligand). Individual protein trajectories were recorded via single-molecule localization microscopy and analyzed using Trackmate [48] and the SMTracker [49] software packages. (**A**) Representative cells of *B. subtilis* and *C. glutamicum* displaying confined (blue) and fast (red) trajectories of DivIVA-Halo. Scale bar 1 µm. (**B**) Plot of the mean-squared displacement of the indicated DivIVA-Halo fusions over time. (**C**) Bubble plot showing single-molecule diffusion rates of the indicated DivIVA fusions. Populations were determined by fitting the probability distributions of the frame-to-frame displacement (jump distance) data of all respective tracks to a three components model (fast mobile, slow mobile, and confined protein populations). (**D**) Determination of average dwell times of the indicated DivIVA fusions via a double exponential decay fit to the survival function (probability of molecules being confined for at least a certain amount of time), here fitted with a two-component model. (**E**) Confinement heat maps of specified DivIVA-Halo fusions, indicating likeliness of presence of confined molecules from low (blue) to high (red) in an averaged cell.

With the structural, functional, and localizational differences between DivIVA from *B. subtilis* and *C. glutamicum* in mind, we next aimed to characterize resulting differences in mobility and confinement in order to understand the molecular details of their individual behavior. To this end, single-molecule localization microscopy (SMLM) in combination with single-particle tracking (SPT) was employed to investigate and compare the respective DivIVA-HaloTag fusions (Figure 2), from here on referred to as DivIVA-Halo. The first striking observation during this comparison was the apparent much higher frequency of fast moving DivIVA molecules in *B. subtilis* (Figure 2A). Using the mean-squared displacement (MSD) as a quantitative indicator of the averaged diffusion speed confirmed this observation, as the resulting diffusion coefficient of DivIVA*_bsu_*-Halo (D = 0.142 µm^2^ s^−1^) was more than 14× larger than for DivIVA*_cgb_*-Halo (D = 0.010 µm^2^ s^−1^, Figure 2B). The slope of the linear fit (Figure 2B) served as a good estimation for the diffusion coefficient for simple Brownian motion of a single population that does not undergo changes in motion. It is, however, inferior to other methods for the identification and quantification of multiple mobility populations or the description of proteins with frequent changes in motion [54], which could both apply to DivIVA due to the ability to bind membrane. Therefore, a jump distance (JD) analysis was performed, identifying three different populations for both DivIVA*_bsu_*-Halo and DivIVA*_cgb_*-Halo (Figure 2C), albeit exposing a similar tendency to the MSD analysis. While DivIVA*_cgb_*-Halo displayed only a minor fast mobile population (1.07%), a great majority of the recorded protein was classified as confined (76.1%) and a significant proportion as slowly diffusive (22.8%). DivIVA*_bsu_*-Halo on the other hand was characterized to possess a large fast mobile population (29.6%) and an even larger slow mobile population (50.7%), while the confined population represents the smallest fraction (19.8%). In line with these results, average dwell time analysis using a two-component model (Figure 2D) revealed a larger proportion of DivIVA*_cgb_*-Halo to be dwelling for a longer time (65 ± 0.84% for 0.73 ± 0.0073 s) when compared to DivIVA*_bsu_*-Halo (49 ± 2.2% for 0.32 ± 0.0053 s). This indicates a much higher probability of DivIVA to dwell or reside in place in *C. glutamicum*, compared to a higher turnover in *B. subtilis*. To be able to identify and compare the intracellular localization of these molecules, a heat map of confined molecules was created (Figure 2E). While the majority of confined DivIVA in *B. subtilis* is likely to be found around the septal site and with lower probability along the lateral membrane and the cell poles, the large majority of confined DivIVA in *C. glutamicum* could be observed at the poles, where it is usually found to form large clusters [30].

### 3.3. Deletion of MinJ, a DivIVA_bsu_ Membrane Integral Partner, Results in an Increased DivIVA Mobility and Decreased Dwelling Time

**Figure 3:**
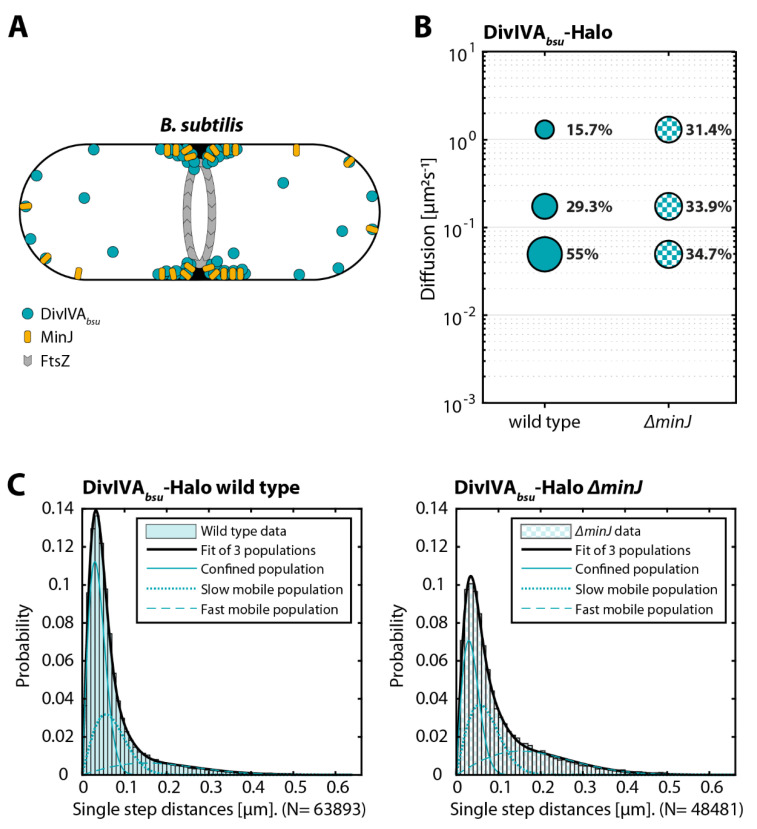
Mobility of DivIVA*_bsu_* is increased in the absence of MinJ. Exponentially growing cells of different *B. subtilis* strains (BHF073 and BHF074) expressing the indicated DivIVA-Halo fusion, respectively, were stained with TMR (HaloTag ligand). Individual protein trajectories were recorded via single-molecule localization microscopy and analyzed using Trackmate [48] and the SMTracker [49] software packages. (**A**) Cartoon of DivIVA and MinJ in a dividing cell. (**B**) Bubble plot showing single-molecule diffusion rates of DivIVA-Halo fusions in indicated strain background. (**C**) Populations were determined by fitting the probability distributions of the frame-to-frame displacement (jump distance) data of all respective tracks to a three-component model (fast mobile, slow mobile, and confined protein populations).

After gaining insights about DivIVA mobility and behavior, we were further interested in the interplay and interdependence of proteins interacting with DivIVA. In *B. subtilis*, MinJ is a direct interaction partner, bridging DivIVA to MinD to be able to (re-)localize the Min system to active sites of division [27,55]. MinJ itself is a transmembrane protein with six predicted transmembrane helices and a PDZ domain, often associated with protein–protein interactions. MinJ is conserved in Bacillus, Listeria, and some Lactobacilli. Deletion of minJ in *B. subtilis* results in divisional defects and thus an increase in cell length and the generation of minicells, likely caused by erroneous divisome disassembly [25,26,27,55]. Furthermore, a recent study from our lab demonstrated the stabilizing effect that MinJ has on DivIVA clusters and mobility via fluorescence recovery after photobleaching (FRAP) experiments [26] (Figure 3A). Therefore, DivIVA was expected to increase its mobility in a Δ*minJ* background. First SPT experiments with a frame length of 20 ms however did not show significant differences (data not shown). As we expected an increase in the fastest population (freely diffusive) and instead found an absolute decrease in tracks, but an increase in intracellular background, we suspected that a shorter frame length of 5 ms would be more appropriate to describe the expected change in mobility. At this strongly decreased frame length, we could indeed see a drastic shift in the mobility population distribution between the wild type and the Δ*minJ* background (Figure 3B,C). While the slow diffusive population only changed mildly (29.3% to 33.9%), the fastest population doubled in size (15.7% to 31.4%), which is even more apparent in the underlying probability distribution of single step distances (Figure 3C). Judging from the data, a large proportion of the confined population instead became mobile in the absence of MinJ (55% to 34.7%, Figure 3B,C), which is in line with previous FRAP results [24]. The dwelling time of DivIVA on the other hand appeared to be almost unaffected by the absence of MinJ, as no significant shifts in population size or dwelling time could be observed (Table 1). The comparably short dwelling times when compared to measurements made at 20 ms frame length (Figure 2D) can be explained by the strong increase in tracks of faster or more mobile molecules that are recorded when using a short frame length (5 ms).

### 3.4. Increased Levels of ParB Results in an Increased DivIVA_cgb_ Mobility and Decreased Dwelling Time

**Figure 4:**
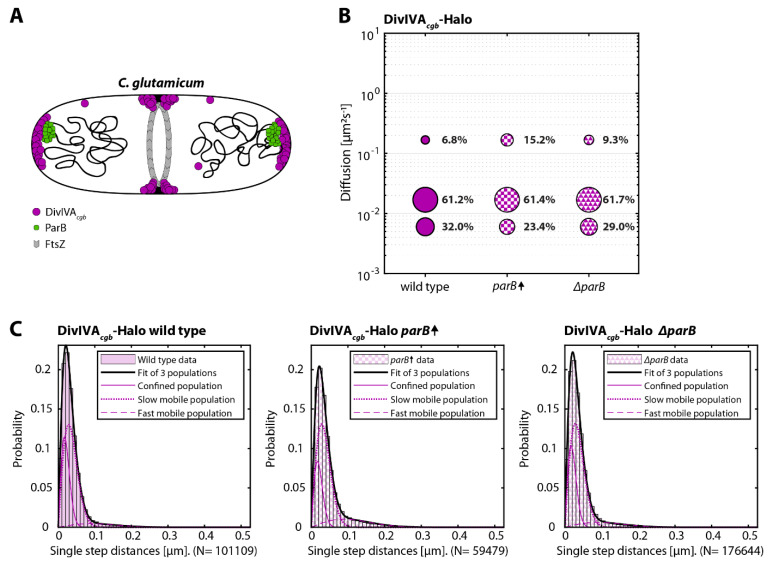
G8, CMG029, and CMG027) expressing the indicated DivIVA-Halo fusion were stained with TMR (HaloTag ligand). Individual protein trajectories were recorded via single-molecule localization microscopy and analyzed using Trackmate [48] and the SMTracker [49] software packages. (**A**) Cartoon of DivIVA and ParB in a dividing cell. (**B**) Bubble plots showing single-molecule diffusion rates of DivIVA-Halo fusions in indicated strain backgrounds. (**C**) Populations were determined by fitting the probability distributions of the frame-to-frame displacement (jump distance) data of all respective tracks to a three components model (fast mobile, slow mobile, and confined protein populations). Overexpression of ParB is indicated by ParB⬆.

As the absence of MinJ resulted in an increased DivIVA*_bsu_* subunits dynamicity, we set forth to characterize DivIVA*_cgb_*-Halo dynamics in *C. glutamicum* strains, expressing three different levels of the centromere-binding protein ParB, which was shown to interact with DivIVA before [34] (Figure 4A).

DivIVA behavior deviated from the one shown for the wild-type strain, both in the presence of increased ParB levels (CMG029) and in the ParB deletion strain (CMG027). Specifically, dwelling time analysis revealed that both mutant strains were characterized by an increase in the short dwelling time population (48.0 ± 1.3% for the ParB overexpression strain and 50.6 ± 1.0 for the Δ*parB* strain compared to the 34.7 ± 0.8% of the wild-type strain) (Table 1).

Similarly, the comparison of the mobile populations obtained via the three-components fit of the jump distance analysis (Figure 4C) showed a shift from the confined population to the fast-mobile population, with the proportion of slowly diffusive molecules remaining relatively constant across all strains (~61%) (Figure 4B). The recorded difference between the mutant strains and the wild type was however more pronounced for the overexpression strain (~8%) compared to the Δ*parB* strain (~3%) (Figure 4B).

We then tested whether we could observe a DivIVA*_cgb_* mobility increase for both the ParB overexpression and ParB deletion strains via FRAP. In order to do so, we compared DivIVA*_cgb_*-mCherry dynamics across the same three genetic backgrounds (wild type–CDC010, ParB overexpression–CDC013, Δ*parB*–CDC012) via FRAP (Figure S1A). The half time recovery distributions obtained for each strain (minimum number of imaged cells of eleven) were tested for normality via the Shapiro–Wilk normality test and for homoscedasticity via the Bartlett test (test of homogeneity of variances). As we could neither observe deviation from normality (*p*-value wild type = 0.1644, *p*-value ParB overexpression = 0.424, *p*-value Δ*parB* = 0.8054) nor observe significant differences in variance across the three distributions (*p*-value = 0.1334), we proceeded to compare the two mutant strains against the wild type via the Dunnett’s test (test for comparing several treatments with a control, alternative hypothesis: true location shift is less than 0). As expected, the half time recovery of DivIVA*_cgb_* in the ParB overexpression strain was significantly faster than the one observed in the wild type (*p*-value ParB overexpression—wild type = 0.042). However, this is not the case for the Δ*parB* strain (*p*-value Δ*parB*—wild type = 0.941). Surprisingly, neither the ParB overexpression nor the Δ*parB* strains showed significant differences when compared to the wild type (*p*-value ParB overexpression—wild type = 0.0845, *p*-value Δ*parB*—wild type = 0.5770).

### 3.5. Cell Shape Drastically Affects DivIVA Behavior in B. subtilis and C. glutamicum

**Figure 5:**
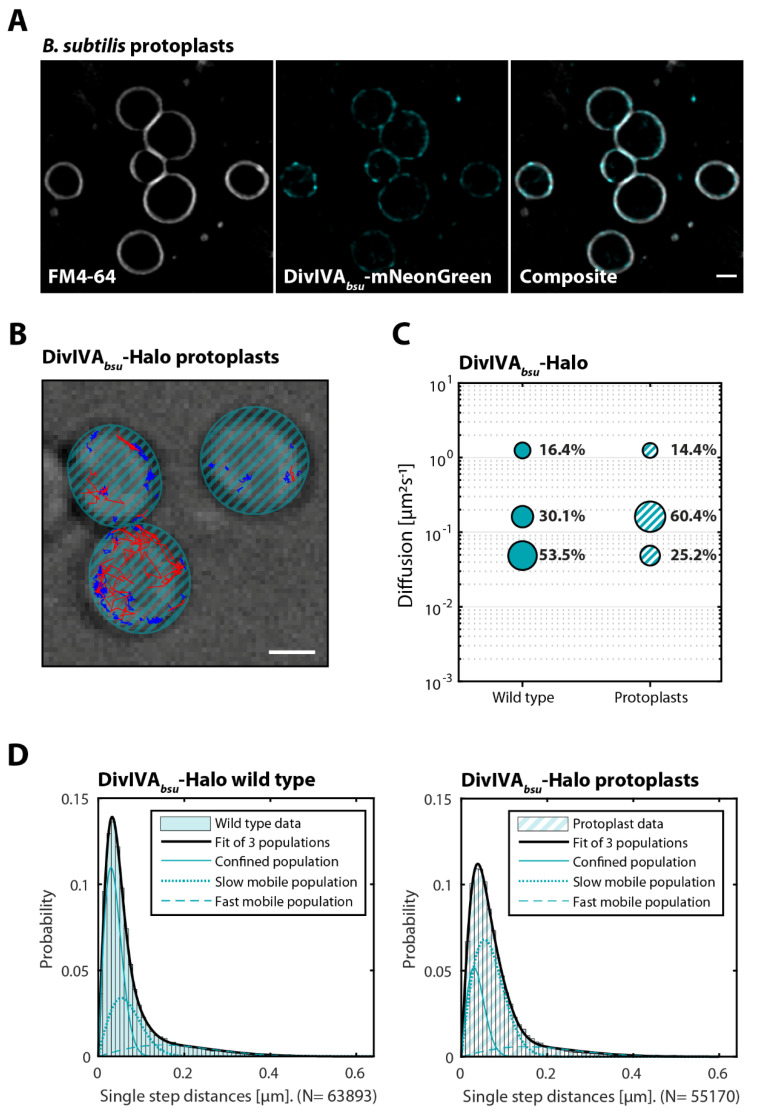
Cell shape drastically affects the mobility of DivIVA*_bsu_*. (**A**) *B. subtilis* protoplasts expressing a DivIVA-mNeonGreen fusion (BHF028) were stained with FM4-64 membrane dye and imaged via SIM^2^. From left to right: FM4-64 membrane dye, DivIVA-mNeonGreen, and composite of both channels. Scale bar 1 µm. Exponentially growing cells/protoplasts of the indicated *B. subtilis* strain (BHF073), respectively, expressing a DivIVA-Halo fusion, were stained with TMR (HaloTag ligand). Individual protein trajectories were recorded via single-molecule localization microscopy and analyzed using Trackmate [48] and the SMTracker [49] software packages. (**B**) Representative cells of *B. subtilis* protoplasts displaying confined (blue) and fast (red) trajectories of DivIVA-Halo. Scale bar 1 µm. (**C**) Bubble plots showing single-molecule diffusion rates of DivIVA-Halo fusions in indicated strain backgrounds. (**D**) Populations were determined by fitting the probability distributions of the frame-to-frame displacement (jump distance) data of all respective tracks to a three components model (fast mobile, slow mobile, and confined protein populations).

**Figure 6:**
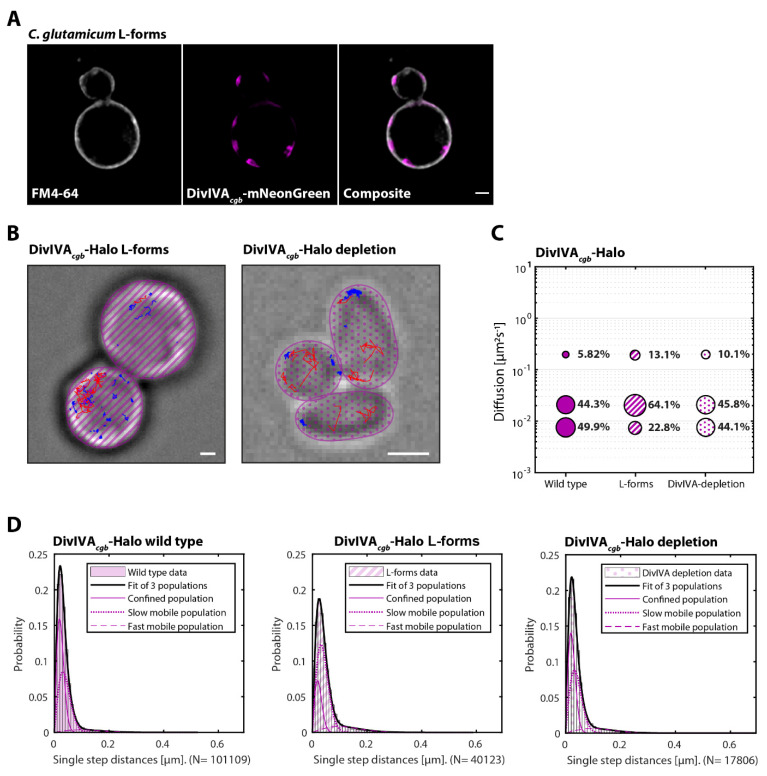
Cell shape drastically affects the mobility of DivIVA*_cgb_*. (**A**) *C. glutamicum* L-forms expressing a DivIVA-mNeonGreen fusion (B4B7) were stained with FM4-64 membrane dye and imaged via SIM^2^. From left to right: FM4-64 membrane dye, DivIVA-mNeonGreen, and composite of both channels. Scale bar 1 µm. Exponentially growing cells/L-forms of *C. glutamicum* strains (B6G8 and B6I1), respectively, expressing a DivIVA-Halo fusion, were stained with TMR (HaloTag ligand). Individual protein trajectories were recorded via single-molecule localization microscopy and analyzed using Trackmate [48] and the SMTracker [49] software packages. (**B**) Representative cells of *C. glutamicum* L-forms and cells with depleted DivIVA-Halo levels displaying confined (blue) and fast (red) trajectories of DivIVA-Halo. Scale bar 1 µm. (**C**) Bubble plots showing single-molecule diffusion rates of DivIVA-Halo fusions in indicated strain backgrounds. Populations were determined by fitting the probability distributions of the frame-to-frame displacement (jump distance) data of all respective tracks to a three components model (fast mobile, slow mobile, and confined protein populations). The dwelling behavior also changed to different extents when comparing DivIVA in both organisms (Table 1). While DivIVA*_bsu_* only displayed a small significant shift from 22.4% ± 0.00 to 27.1% ± 0.00 in the short dwelling time population, it considerably increased from 34.7 ± 0.84% to 60.0 ± 1.06% for DivIVA*_cgb_*. (**D**) Populations were determined by fitting the probability distributions of the frame-to-frame displacement (jump distance) data of all respective tracks to a three components model (fast mobile, slow mobile, and confined protein populations).

In previous studies, DivIVA has been demonstrated to autonomously localize to regions of negative curvature [22,23], serving as a spatial cue for the localization of different interaction partners, depending on the organism [52]. After demonstrating that interaction partners affect the mobility of DivIVA, we were next interested in the impact of changes in the respective cell shapes, specifically a reduction of negative curvature. Henceforth, SPT experiments were extended to spherical cells that lost their rod shape, namely protoplasts of *B. subtilis* and L-forms of *C. glutamicum*, respectively (representative illustration in Figure 5B and Figure 6B). Briefly, in the presence of homogeneous membrane curvature, DivIVA*_bsu_* did not appear to have specific affinity for any membrane region (Figure 5A). Contrary to that, DivIVA*_cgb_* retained the ability to form the quasi-homogeneous clusters that would normally be observed at the polar regions (Figure 6A). Notably, the change of shape also had an impact on the mobility of DivIVA, specifically on the slow diffusive and confined populations. In both organisms, the slow mobile population increased drastically (30.1% to 60.4% DivIVA*_bsu_* and 44.3% to 64.1% DivIVA*_cgb_*), while the confined population decreased in size by a similar extent (Figure 5C,D and Figure 6C,D). At the same time, the fast-mobile population of DivIVA*_cgb_* increased substantially from 5.82% to 13.1%, while it decreased by only 2% in *B. subtilis*.

A similar, yet not identical, pattern can be observed when comparing the half time recovery of DivIVA*_cgb_* in L-forms compared to wild type cells (Figure S1B). As the Shapiro–Wilk normality test revealed that the half time recovery for DivIVA*_cgb_* clusters in L-forms does not follow a normal distribution (*p*-value wild type = 0.1644, *p*-value, *p*-value L-forms = 1.232 × 10^−6^), a comparison between the conditions was performed via the Wilcoxon rank-sum test with continuity correction (alternative hypothesis: true location shift is less than 0). The test confirmed that DivIVA*_cgb_* dynamics are enhanced in L-forms (*p*-value = 0.005196).

### 3.6. Depletion of DivIVA Concentration Leads to Increased DivIVA Mobility 

We showed that changing the shape of a cell to a sphere causes an increase in DivIVA diffusion in both *B. subtilis* and *C*. *glutamicum* (Figure 5C and Figure 6C). No parallel between the two can however be drawn when observing the changes in protein localization (Figure 5A and Figure 6A). We therefore decided to challenge DivIVA*_cgb_* protein dynamics and localization by depleting its levels via the CRISPRi system developed by the Bai lab [39]. As previously shown [30], the depletion of DivIVA*_cgb_* has dramatic effects on cellular growth and morphology (Figure S2B). Where wild type cells are characterized by a club-like shape, with cell wall synthesis and DivIVA being confined to polar and septal regions (Figure S2A), the depletion of DivIVA resulted in a tendency toward coccoid morphology and loss of both DivIVA and cell wall synthesis defined localizations (Figure S2B). Interestingly, in the DivIVA depleted cells, peptidoglycan synthesis appeared uncoupled from DivIVA, and hence, DivIVA was apparently not required for activation of cell wall synthetic clusters. 

Strikingly, a comparison of the single step distances obtained for the DivIVA*_cgb_* depletion strain (B6I1) with those obtained for the wild-type strain (B6G8) revealed a ~5% population shift between the confined and the fast-mobile population, with little variation in the proportion of slowly diffusive molecules (Figure 6C). This change diverged from the one obtained for the L-forms, where the shift was concentrated toward the slowly diffusive population (Figure 6C).

Differences in the dynamics of DivIVA*_cgb_* between the wild-type strain (B6G8) and the depletion strain (B6I1) extended to the analysis of the dwelling time. Specifically, dwelling time analysis revealed that the depletion strain was characterized by an ~11% increase in the short dwelling time population (46.2 ± 1.3% for depletion strain compared to the 34.7 ± 0.8% of the wild-type strain) (Table 1).

## 4. Discussion

**Figure 7:**
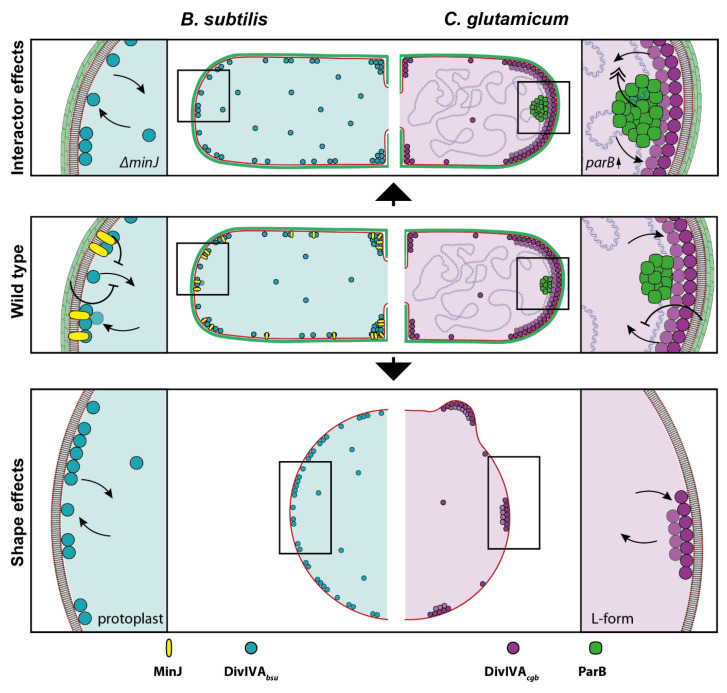
Model describing DivIVA dynamics and localization in *B. subtilis* and *C. glutamicum*. (**Central panel**): In the rod-shaped, wild type cells, DivIVA is localized to the cell poles in *C. glutamicum* (magenta) with only very little free diffusive molecules. At the onset of cell division, DivIVA starts to accumulate at the septum. The restricted area at the cell pole reduces the mobility of DivIVA*_cgb_* and leads to stable accumulation of protein clusters at the cell poles. In *B. subtilis* (cyan) DivIVA dynamics are also influenced by the narrow region of the cell poles. Additionally, MinJ stabilizes DivIVA clusters. In contrast to the *C. glutamicum* protein, DivIVA*_bsu_* is more mobile with a larger diffusive population, allowing localization of DivIVA*_bsu_* also along the lateral cell membrane. Deletion of MinJ leads to an increase in mobility of DivIVA*_bsu_* (**upper panel**). In contrast, an increase of ParB concentration in *C. glutamicum* increases the mobility of DivIVA*_cgb_*. Loss of rod-shape morphology increases the mobility of DivIVA in *C. glutamicum* and *B. subtilis*. While DivIVA*_bsu_* distributes in small clusters evenly along the protoplast membrane, DivIVA*_cgb_* still forms large, defined clusters that may even be able to deform the membrane and allow new pole formation (**lower panel**).

Bacterial cells use scaffold proteins to localize protein complexes or macro-molecules, such as the chromosome, to specific places within the cell. Cells basically employ two mechanisms for this spatiotemporal organization. Classical cytoskeletal proteins, for instance actin or tubulin, form dynamic structures in which the dynamic behavior is regulated through direct nucleotide hydrolysis. In bacterial cells, MreB/MreB-like proteins use ATP hydrolysis to form dynamic filaments that position the cell wall synthesis machinery necessary for cell elongation. Furthermore, localization of the cell wall synthesis machinery during cytokinesis has been shown to rely on the GTP hydrolysis-dependent formation of FtsZ (a tubulin homolog) dynamic filaments [56]. Nucleotide-independent cytoskeletal scaffold proteins, in contrast, have been generally considered as rigid scaffolds [21]. Biophysical cues such as membrane curvature have been implicated in their positioning [23,57]. However, their dynamics have not been studied in great detail. Initial single-molecule tracking analysis of PopZ, the polar scaffold for chromosome orientation in *C*. *crescentus*, revealed two subpopulations of PopZ [58]. The majority of the PopZ was shown to localize fixed to the cell poles, while a mobile fraction was diffusive within the cytoplasm. Therefore, a diffusion capture mechanism has been proposed, in which PopZ molecules subsequently become sequestered to the cell poles [20,58]. Similar to PopZ, the coiled-coil protein DivIVA was thought to stably localize to the cell poles and to active division sites [22,23,59]. SIM imaging and FRAP imaging revealed that in *B. subtilis*, DivIVA rings at the division site are stable structures that appear to collapse into patches that remain at the cell poles. These DivIVA patches were thought to be relatively inert and to not incorporate newly synthesized protein [60]. We have recently provided evidence that the *B. subtilis* DivIVA is more dynamic than initially thought and that it dynamically relocalizes from the cell poles to active septa with the exchange of molecules between clusters [24,26]. Here, we aimed at a more precise analysis of DivIVA dynamics using single-molecule tracking. Furthermore, we wanted to compare DivIVA dynamics in different species, each belonging to one of the two main DivIVA subgroups [52,61], to unravel whether their molecular dynamics may explain the various functions that these scaffold proteins have in bacteria. Finally, we summarized the main findings about DivIVA dynamics and localization in *B. subtilis* and *C. glutamicum* in a model (Figure 7).

Using SPT and FRAP, we show that DivIVA from *B. subtilis* and *C. glutamicum* is not an inert scaffold that solely acts as a landmark protein. Rather, we show that DivIVA is a dynamic protein that can be observed in three distinct mobility populations. The largest fraction of DivIVA is confined and resides at regions with high membrane curvature, such as the cell poles or the septal region (Figure 7, central panel). This is in accord with all previous localization studies. The preference of DivIVA for a curved membrane has been explained with a molecular bridging model [23]. Stabilization of DivIVA by multivalent interactions with the membrane and between DivIVA molecules is the reason for the confined localization. However, we show here that dynamics of DivIVA molecules from *C. glutamicum* differ from those in *B. subtilis*. DivIVA in both species shows three distinct mobility populations with specific diffusion constants. We termed the slowest population confined and the more mobile populations slow and fast mobile fraction. Interestingly, a comparison of the two DivIVA with the same imaging conditions reveal that DivIVA*_bsu_* have generally ten times bigger MSD than their DivIVA*_cgb_* counterpart. Furthermore, the dwell-time of the *C. glutamicum* DivIVA is much larger. Together, this leads to a more confined localization of DivIVA to the cell poles in *C. glutamicum*. In comparison, the *B. subtilis* DivIVA is far more mobile. The MSD is higher, and the dwell-time of DivIVA*_bsu_* is smaller compared to DivIVA*_cgb_*. These differences are not explainable by the larger molecule size of DivIVA*_cgb_*, but rather point to more stable interactions between *C. glutamicum* DivIVA molecules either with each other, with the membrane, or both. This is in line with the function of DivIVA in actinobacteria to localize the apical cell wall synthetic machinery [31,32] and to tether the origin of replication to the cell poles [9,34]. The *B. subtilis* DivIVA, in contrast, needs to quickly relocalize to the site of septation [26], and hence, a significant amount of protein is always found at the lateral sites, shuttling between the poles and midcell while probing the cell for negative curvature. The molecular differences in the length of the coiled-coil region and the membrane-binding domain alter the protein dynamics and, consequently, the subcellular localization. 

The main role of cytoskeletal scaffolds is the recruitment of other proteins, or protein complexes, to defined cellular regions [57]. Currently, it is unclear whether these interaction partners modulate the dynamics of the scaffold. We show here two examples of how interacting proteins modulate DivIVA dynamics in opposite directions. In *C. glutamicum*, ParB binds to DivIVA [34], thereby localizing the chromosome in a longitudinal fashion with the *oriC* facing towards the cell poles. This organization gives rise to an important, unsolved question: how are the replicated origins separated with one origin segregating away while the other remains at the pole? Our data show that in *C. glutamicum* the ParB protein directly modulates DivIVA dynamics. Although the effect is not drastic, DivIVA mobility is increased in the presence of high levels of ParB. This could help to weaken the stable DivIVA clusters and the interaction with ParB, allowing the segregation of one origin during the cell cycle [9]. Interestingly, while the absence of ParB also seems to affect DivIVA dynamics, it does not have the opposite effect of ParB overexpression. If present, the hypothesized stabilizing effect of a ParB deletion could, in fact, be hidden by the pleiotropic effect that it has on *C. glutamicum* cells, such as chromosome segregation and cell morphology. In contrast, the *B. subtilis* DivIVA that is more mobile is stabilized by its interaction partner MinJ. In a MinJ deletion strain, the confined population of DivIVA*_bsu_* is significantly reduced, and the fast mobile, free diffusible fraction largely increased. Thus, MinJ exerts an inhibitory effect on DivIVA*_bsu_* dynamics, which is important to stabilize the DivIVA cluster at the site of septation. Consequently, loss of MinJ leads to a decrease in stable accumulations of DivIVA [26,27]. The molecular function of DivIVA as scaffold is therefore modulated by other protein factors and tailored towards a specific molecular task that either renders a more stable complex dynamic or a more dynamic complex stable in response to protein–protein interactions (Figure 7, upper panel). 

Our data also reveal a remarkable influence of cell shape on DivIVA dynamics (Figure 5, Figure 6 and Figure 7, lower panel). In both *B. subtilis* and *C. glutamicum*, we observed that DivIVA dynamics were increased when the rod-shaped morphology was lost. In *C. glutamicum*, the confined population is halved in round cells. The slow and fast diffusive populations are significantly increased, showing the overall increased DivIVA dynamics when the rod-shaped morphology is lost. Thus, the binding of DivIVA clusters to a restricted pole area with larger membrane curvature greatly stabilized the protein complexes. This finding is well in accord with the cellular role of DivIVA in actinobacteria, where it recruits the cell wall synthetic proteins and thus spatially governs apical growth. The fact that DivIVA still forms large clusters that are defined and well separated along the membrane in L-form cells has important implications. Specifically, it means that these clusters are still able to recruit the cell wall synthesis machinery, therefore allowing for the establishment of a new growth zone. This quickly sets a polarity axis where the size of the DivIVA cluster defines the cell pole area. As the construction of novel cell wall material is coupled with the tethering of the chromosome, a rod-shaped morphology, and ultimately a viable cell, it can be quickly regained from round cells. In case of the *B. subtilis* DivIVA, loss of cell shape also leads to an increased protein mobility, indicating a conserved mechanism. However, such as in rod-shaped cells, DivIVA*_bsu_* forms multiple smaller clusters that are spread all over the cell membrane in protoplasts and do not accumulate into large clusters. This pattern is comparable with the DivIVA localization that is observed along the lateral cell membrane in rod-shaped cells [26]. Dynamics of *C. glutamicum* DivIVA is furthermore influenced by the protein concentration. Depletion of DivIVA expression in a CRISPRi approach increases the fast-diffusive population about two-fold. Thus, high protein concentrations stabilize the cluster formation. Our data reveal a nice example of cellular self-organization that helps to maintain a uniform cell morphology (Figure 6). DivIVA dynamics is directly affected by cell shape, and in turn, DivIVA recruits the machinery that establishes the cell shape by synthesizing the peptidoglycan exoskeleton. The negative feedback of the cell shape on the DivIVA dynamics stabilizes the system and allows for the establishment of a constant morphology. 

This is particularly important in actinobacteria since these bacteria lack the MreB scaffolded elongation machinery. MreB filaments bind to curved membranes, and their translocation is determined by the membrane curvature to reinforce the rod shape [62,63]. Apparently, self-organization principles evolved to maintain a robust morphology of bacterial cells, and these systems can react upon alterations in cell shape. Feedback loops are often found in biology to maintain robust homeostasis. The effect of feedback loops is best known in gene expression. The data presented here show how feedback loops allow constant cell shape. It is intriguing to see how cellular self-organization is coupled to biophysical cues and how conserved proteins have evolved to allow for specific functions, despite being able to fulfill their initial role. 

## Figures and Tables

**Table 1:** DivIVA dwelling time analysis.

Strain	Շ1 [s]	Շ1%	Շ2 [s]	Շ2%
*C. glutamicum*				
Wild type (B6G8)	0.19 ± 0.0040 s	34.7 ± 0.84%	0.76 ± 0.0073 s	65.3 ± 0.84%
ParB⬆ * (CMG029)	0.20 ± 0.0051 s	48.0 ± 1.34%	0.83 ± 0.0160 s	52.0 ± 1.34%
Δ*parB* (CMG027)	0.20 ± 0.0037 s	50.6 ± 1.00%	0.84 ± 0.0130 s	49.4 ± 1.00%
Wild type L-forms (B6G8)	0.19 ± 0.0033 s	60.0 ± 1.06%	0.86 ± 0.0190 s	40.0 ± 1.06%
DivIVA depletion (B6I1)	0.22 ± 0.0050 s	46.2 ± 1.33%	0.77 ± 0.0130 s	53.8 ± 1.33%
*B. subtilis*				
Wild type (BHF073)	0.036 ± 0.0000 s	19.3 ± 0.00%	0.11 ± 0.0024 s	80.7 ± 0.00%
Δ*minJ* (BHF074)	0.036 ± 0.0000 s	22.4 ± 0.00%	0.11 ± 0.0025 s	77.6 ± 0.00%
Wild type protoplasts (BHF073)	0.036 ± 0.0000 s	27.1 ± 0.00%	0.10 ± 0.002 s	72.9 ± 0.00%

* ⬆ indicates increased expression level of parB.

## Data Availability

Not applicable.

## Supplementary Materials

The following supporting information can be downloaded at: https://www.mdpi.com/article/10.3390/genes13020278/s1, Figure S1: FRAP analysis reveals that DivIVA*_cgb_* cluster dynamics is affected by ParB and cell shape, Figure S2: DivIVA depletion affects cell shape and localization of cell wall synthesis, Table S1: Oligonulceotids, Table S2: Plasmids, Table S3: Strains, Table S4: Media and Buffers.
