# Supplementary material for: Subcellular Dynamics of a Conserved Bacterial Polar Scaffold Protein
Source: Genes (Basel). 2022 Jan 30;13(2):278. doi: 10.3390/genes13020278 (PMC8872289; doi:10.3390/genes13020278)
Supplement: Supplementary file 1 [file genes-13-00278-s001.zip › genes-1541061-supplementary.pdf]

Supplemental Material

Subcellular dynamics of a conserved bacterial polar scaffold protein

Giacomo Giacomelli[1,2#], Helge Feddersen[1,2#], Feng Peng[1], Gustavo Benevides Martins[2], Manuela Grafemeyer[1], Fabian Meyer[1,2], Benjamin Mayer[3,4], Peter L. Graumann[3,4] and Marc Bramkamp[1,2,5]*

[1] Institute for General Microbiology, Christian-Albrechts-University Kiel, Am Botanischen Garten 1-9, 24118 Kiel, Germany

[2] Ludwig-Maximilians-University Munich, Faculty of Biology, Großhaderner Straße 2-4, 82152 Planegg-Martinsried, Germany

[3] SYNMIKRO, LOEWE-Zentrum für Synthetische Mikrobiologie, Karl von Frisch Straße 14, D-35043, Marburg, Germany

[4] Fachbereich Chemie, Universität Marburg, Hans-Meerwein-Straße 4, D-35032, Marburg, Germany.

[5] Central Microscopy Facility, Christian-Albrechts-University Kiel, Am Botanischen Garten 1-9, 24118 Kiel, Germany

Running title: DivIVA dynamics in bacteria

# These authors contributed equally

*To whom correspondence should be addressed: Marc Bramkamp, Christian-Albrechts-University Kiel, Institute for General Microbiology, Am Botanischen Garten 1-9, 24118 Kiel, Germany, Email: bramkamp@ifam.uni-kiel.de; Phone: +49 (0)431-880-4341; Telefax: +49(0)431-880-2198, Twitter: @BramkampLab

Table S1: Oligonucleotides used for strain construction

Restriction sites are underlined, sgRNA annealing is shown in bold.

Name	Sequence (5´-3´)	Restriction site
SalI-5´Halo-F	CGATGC GTCGAC ATG GAAATCGGTACTGGCTTTCC	SalI
XbaI-TAA-3´Halo-R	CGATGC TCTAGA TTA GGAAATCTCCAGAGTAGACAGCC	XbaI
sgRNA-divIVA-F	TGTGG**AGCCTTCTGAGCGGCCTTGG**G	-
sgRNA-divIVA-R	AAAAC**CCAAGGCCGCTCAGAAGGCT**C	-
HF0061	GTC GGTCTC AACTAGAATTCGTAATCATGGTCATAGCTG	BsaI
HF0062	CTC GGTCTC ATCGGAAGCTTGGCACTGGCC	BsaI
G32	CTA GGTCTC TCCGAGAATTCCTAGCCCAAGTCAG	BsaI
G33	TAT GGTCTC CCTCCTTCCTTTTCCTCAAA	BsaI
G34	CTA GGTCTC TCCGAGAATTCCTAGCCCAAGTCAG	BsaI
G35	TAT GGTCTC CCTCCTTCCTTTTCCTCAAA	BsaI
G36	GGA GGTCTC TTTCGGGGTGAAAGGATGTACTTA	BsaI
G37	CAC GGTCTC CCATTTAATTGAGAGAAGTT	BsaI
HF0211	AAT GGTCTCT GGAGGGATGGAAATCGGTACTGG	BsaI
HF0212	TTT GGTCTC GCGAATTAGGAAATCTCCAGAGTAGAC	BsaI

Table S2: Plasmids

Name	Features	Source
	C. glutamicum	
pSG-dCas9	Ptac, lacIq, repA, ori PUC, dCas9, sgRNA scaffold,Kmr	[1]
pSG-dCas9_sgRNA-divIVA	Ptac, lacIq, repA, ori PUC, dCas9, *diVIVA*-sgRNA,Kmr	This study
pk19mobsacB	Integration vector, ori pUC, Kmr, mob sacB	[2]
pk19mobsacB-divIVA-mNeonGreen	Integration vector, ori pUC, Kmr, mob sacB, *divIVA-mNeonGreen*	[3]
pk19mobsacB-divIVA-HaloTag	Integration vector, ori pUC, Kmr, mob sacB, *divIVA-HaloTag*	This study
pk19mobsacB-divIVA-mCherry	Integration vector, ori pUC, Kmr, mob sacB, *divIVA-mCherry*	[4]
pk19mobsacB- ΔparB	Integration vector, ori pUC, Kmr, mob sacB, deletion of *parB*	[5]
pEKEx2-ParB-eCFP	Ptac lacIq pBL1 oriVC.g. pUC18 oriVE.c., ParB+-CFP	[5]

B. subtilis		
pUC18mut	pUC18 with mutated BsaI site in bla	[6]
pHF29	pUC18mut divIVA-Halo-aad9-divIVAdown	This study
pJet1.2 Tev-Halo tag	pJet1.2 Tev-HaloTag, Ampr	Addgene [7]

Table S3: Strains

**Name**	**Genotype**	**Source**
C. glutamicum		
RES167_IsceIrs	*C. glutamicum RES167 adhA::adhA_sw-IsceI_rs* (Restriction-deficient mutant, otherwise considered wild-type)	This study
B4B7	RES167_IsceIrs derivative, *divIVA::divIVA-mNeonGreen*	This study
B6G8	RES167_IsceIrs derivative, *divIVA::divIVA-HaloTag*	This study
CMG027	B6G8 derivative, *ΔparB*	This study
B6I1	B6G8 derivative, pSG-dcas9_sgRNA-divIVA	This study
CMG029	B6G8 derivative, pEKEx2-ParB-CFP	This study
RES167	*C. glutamicum RES167* (Restriction-deficient mutant, otherwise considered wild-type)	[8]
CDC010	RES167 derivative, *divIVA::divIVA-mCherry*	[4]
CDC012	CDC010 derivative, *ΔparB*	[4]
CDC013	CDC010 derivative, pEKEx2-ParB-eCFP	[4]
MB001	*C. glutamicum MB001*	[9]
CPF004	MB001 derivative, *divIVA::divIVA-mCherry*	This study
CPF005	CPF004 derivative, pSG-dcas9_sgRNA-divIVA	This study
B. subtilis		
168	trpC2; *B. subtilis* wild type 168	Laboratory collection
RD021	168 derivative, *minJ::tet*	[10]
BHF028	168 derivative, *divIVA::divIVA-mNeonGreen-aad9*	[11]
BHF073	168 derivative, *divIVA::divIVA-HaloTag-aad9*	This study
BHF074	RD021 derivative, *divIVA::divIVA-HaloTag-aad9*	This study

E. coli		
NEB5α	*fhuA2 Δ(argF-lacZ)U169 phoA glnV44 Φ80 Δ(lacZ)M15 gyrA96 recA1 relA1 endA1 thi-1 hsdR17*	New England Biolabs

Table S4: Media and Buffers

Name	Recipe	Source
Media		
MSM (2X)	1 M sucrose, 40 mM Maleic acid and 40 mM $MgCl_2$. pH 7	[12]
CGXII (2X)	per liter: 40 g $(NH_4)_2SO_4$, 10 g Urea, 2 g KH_2PO_4, 2 g K_2HPO_4, 0.5 g $MgSO_4$ x 7 H_2O, 84 g MOPS, 20mg $CaCl_2$, 20 mg $FeSO_4$ x $7H_20$, 20 mg $MnSO_4xH_2O$, 2 mg $ZnSO_4$ x $7H_2O$, 0.4 mg $CUSO_4$, 0.04 mg of NiCl2 x $6H_2O$, 0.4 mg biotin (pH 7), 0.06 mg protocatechuic acid and 80 g glucose	[13]
MSM/CGXII	Mix equal amounts of CGXII (2X) and MSM (2X)	[14]
MD (2X)	per liter: 21.4 g K_2HPO_4, 12 g KH_2PO_4, 2 g Na_3 citrate, 40 g glucose, 40 g l-tryptophan, 40 g ferric ammonium citrate, 50 g l-aspartate, 0.72 g $MgSO_4$ and 2 g casamino acids	[15]
MSM/MD	Mix equal amounts of MD (2X) and MSM (2X)	This study
NB and NA	Nutrient broth/agar	-
BHI	Brain-Heart Infusion medium/agar	-
Buffers		
PBS	137 mM NaCl, 2.7 mM KCl, 10 mM Na_2HPO_4 and 1.8 mM KH_2PO_4	-
TSEMS	50 mM Tris pH 7.4, 50 mM NaCl, 10 mM EDTA and 0.5 M sucrose	[16]

Supplemental Material References

1. Peng, F.; Wang, X.; Sun, Y.; Dong, G.; Yang, Y.; Liu, X.; Bai, Z. Efficient gene editing in *Corynebacterium glutamicum* using the CRISPR/Cas9 system. *Microb Cell Fact* **2017**, *16*, 201, doi:10.1186/s12934-017-0814-6.
2. Schafer, A.; Tauch, A.; Jager, W.; Kalinowski, J.; Thierbach, G.; Puhler, A. Small mobilizable multi-purpose cloning vectors derived from the *Escherichia coli* plasmids pK18 and pK19: selection of defined deletions in the chromosome of *Corynebacterium glutamicum*. *Gene* **1994**, *145*, 69-73, doi:10.1016/0378-1119(94)90324-7.
3. Schubert, K.; Sieger, B.; Meyer, F.; Giacomelli, G.; Bohm, K.; Rieblinger, A.; Lindenthal, L.; Sachs, N.; Wanner, G.; Bramkamp, M. The Antituberculosis Drug Ethambutol Selectively Blocks Apical Growth in CMN Group Bacteria. *mBio* **2017**, *8*, doi:10.1128/mBio.02213-16.

4. Donovan, C.; Sieger, B.; Kramer, R.; Bramkamp, M. A synthetic *Escherichia coli* system identifies a conserved origin tethering factor in Actinobacteria. *Mol Microbiol* **2012**, *84*, 105-116, doi:10.1111/j.1365-2958.2012.08011.x.
5. Donovan, C.; Schwaiger, A.; Kramer, R.; Bramkamp, M. Subcellular localization and characterization of the ParAB system from *Corynebacterium glutamicum*. *J Bacteriol* **2010**, *192*, 3441-3451, doi:10.1128/JB.00214-10.
6. Feddersen, H.; Wurthner, L.; Frey, E.; Bramkamp, M. Dynamics of the *Bacillus subtilis* Min System. *mBio* **2021**, *12*, doi:10.1128/mBio.00296-21.
7. Diebold-Durand, M.L.; Burmann, F.; Gruber, S. High-Throughput Allelic Replacement Screening in *Bacillus subtilis*. *Methods Mol Biol* **2019**, *2004*, 49-61, doi:10.1007/978-1-4939-9520-2_5.
8. Tauch, A.; Kirchner, O.; Loffler, B.; Gotker, S.; Puhler, A.; Kalinowski, J. Efficient electrotransformation of *Corynebacterium diphtheriae* with a mini-replicon derived from the *Corynebacterium glutamicum* plasmid pGA1. *Curr Microbiol* **2002**, *45*, 362-367, doi:10.1007/s00284-002-3728-3.
9. Baumgart, M.; Unthan, S.; Ruckert, C.; Sivalingam, J.; Grunberger, A.; Kalinowski, J.; Bott, M.; Noack, S.; Frunzke, J. Construction of a prophage-free variant of *Corynebacterium glutamicum* ATCC 13032 for use as a platform strain for basic research and industrial biotechnology. *Appl Environ Microbiol* **2013**, *79*, 6006-6015, doi:10.1128/AEM.01634-13.
10. Bramkamp, M.; Emmins, R.; Weston, L.; Donovan, C.; Daniel, R.A.; Errington, J. A novel component of the division-site selection system of *Bacillus subtilis* and a new mode of action for the division inhibitor MinCD. *Mol Microbiol* **2008**, *70*, 1556-1569, doi:10.1111/j.1365-2958.2008.06501.x.
11. Stockmar, I.; Feddersen, H.; Cramer, K.; Gruber, S.; Jung, K.; Bramkamp, M.; Shin, J.Y. Optimization of sample preparation and green color imaging using the mNeonGreen fluorescent protein in bacterial cells for photoactivated localization microscopy. *Sci Rep* **2018**, *8*, 10137, doi:10.1038/s41598-018-28472-0.
12. Mercier, R.; Kawai, Y.; Errington, J. General principles for the formation and proliferation of a wall-free (L-form) state in bacteria. *Elife* **2014**, *3*, doi:10.7554/eLife.04629.
13. Keilhauer, C.; Eggeling, L.; Sahm, H. Isoleucine synthesis in *Corynebacterium glutamicum*: molecular analysis of the *ilvB-ilvN-ilvC* operon. *J Bacteriol* **1993**, *175*, 5595-5603, doi:10.1128/jb.175.17.5595-5603.1993.
14. Martins, G.B.; Giacomelli, G.; Goldbeck, O.; Seibold, G.M.; Bramkamp, M. Substrate-dependent cluster density dynamics of *Corynebacterium glutamicum* phosphotransferase system permeases. *Mol Microbiol* **2019**, *111*, 1335-1354, doi:10.1111/mmi.14224.
15. Anagnostopoulos, C.; Spizizen, J. Requirements for Transformation in *Bacillus subtilis*. *J Bacteriol* **1961**, *81*, 741-746, doi:10.1128/jb.81.5.741-746.1961.
16. Bohm, K.; Giacomelli, G.; Schmidt, A.; Imhof, A.; Koszul, R.; Marbouty, M.; Bramkamp, M. Chromosome organization by a conserved condensin-ParB system in the actinobacterium *Corynebacterium glutamicum*. *Nat Commun* **2020**, *11*, 1485, doi:10.1038/s41467-020-15238-4.

Supplemental Material Figures

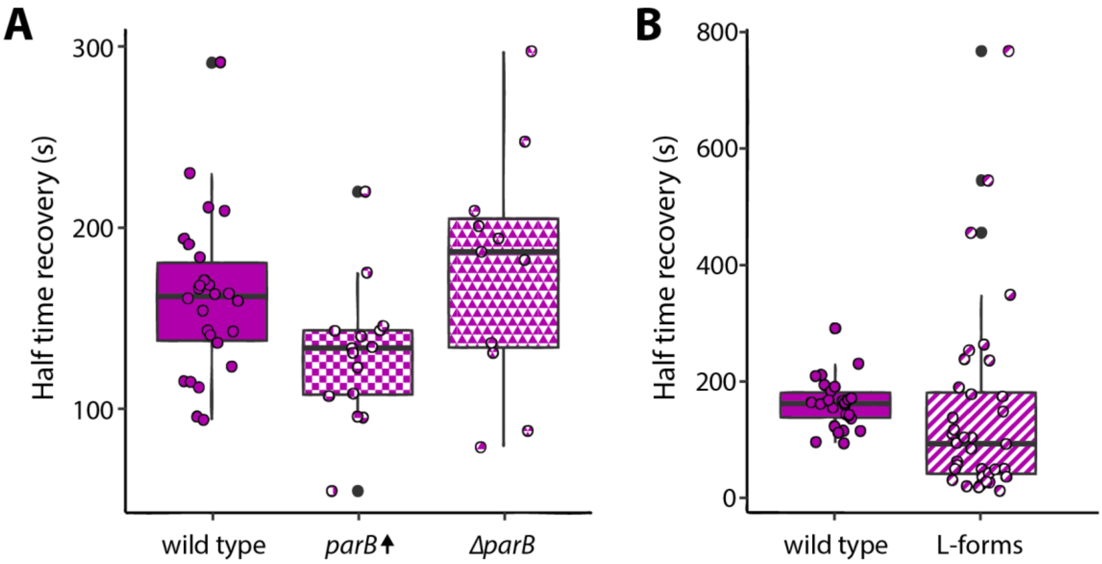

Figure S1: FRAP analysis reveals that DivIVA*cgb* cluster dynamics is affected by ParB and cell shape. (A) The half time recovery for DivIVA*cgb* polar clusters was determined for *C. glutamicum* strains expressing DivIVA-mCherry in the respective genetic background (CDC010, CDC012 and CDC013). **(B)** The half time recovery for DivIVA*cgb* clusters was determined for *C. glutamicum* cells/L-forms expressing DivIVA-mCherry in the respective genetic background (CDC010).

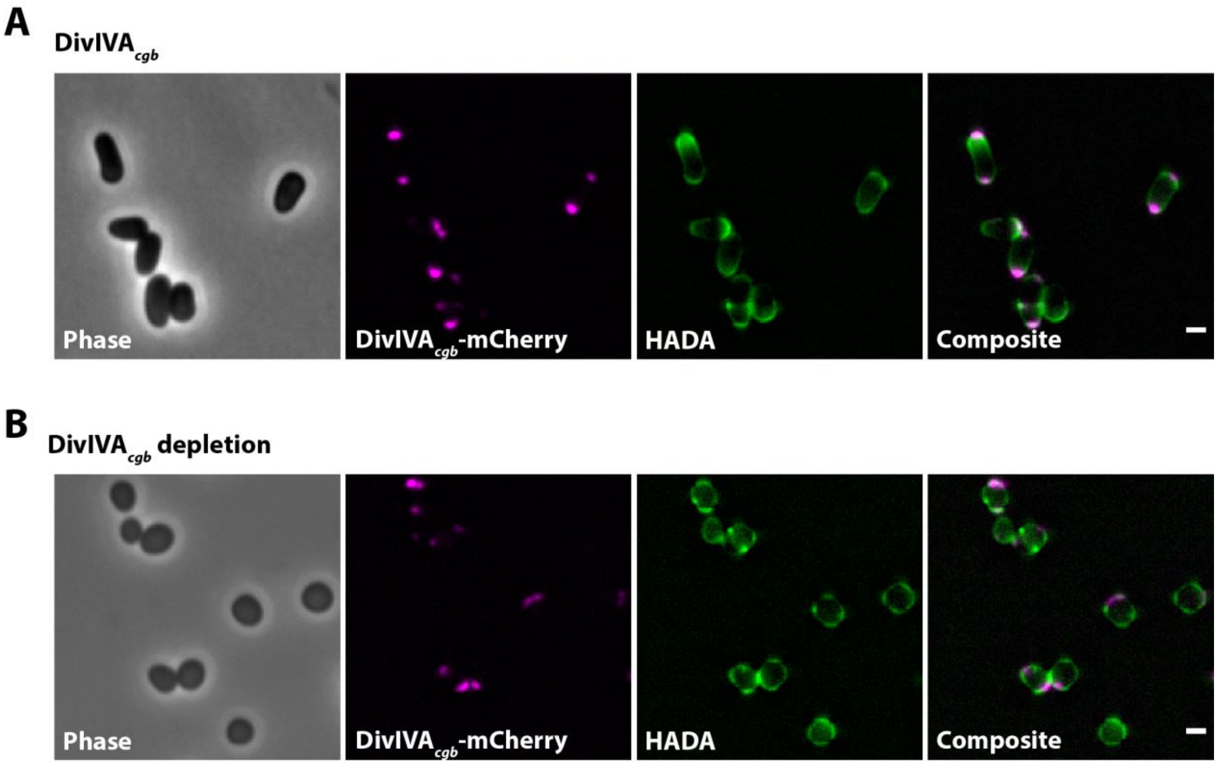

Figure S2: DivIVA depletion affects cell shape and localization of cell wall synthesis. *C. glutamicum* cells (CPF005) with wild type **(A)** or depleted **(B)** DivIVA-mCherry levels were shortly stained (5 minutes) with HADA in order to visualize sites of nascent cell wall production. From left to right: phase contrast, DivIVA$_{cgb}$-mCherry, HADA and composite between DivIVA$_{cgb}$-mCherry and HADA fluorescence. Scale bar 1µm.
